# Priorities for rheumatic and musculoskeletal disease research in Ireland

**DOI:** 10.1186/s41927-022-00285-9

**Published:** 2022-08-11

**Authors:** Emma R. Dorris, Stacey Grealis, Karmen Kegl, Norelee Kennedy, Louise Larkin, Brian Lynch, Ailis Moran, Justine O’Brien, Stephanie Skeffington, Kayleigh Slater, Rebecca Ward, Allison Willett

**Affiliations:** 1 University College Dublin, Belfield, Dublin 04, D04V1W8 Ireland; 2 Mayo, Ireland; 3 Dublin, Ireland; 4 Discipline of Physiotherapy, School of Allied Health, Faculty of Education and Health Sciences, University of Limerick and Health Research Institute (HRI), University of Limerick, Limerick, Ireland; 5 Health Research Institute (HRI), University of Limerick, Limerick, Ireland; 6 Arthritis Ireland, Dublin 02, Ireland; 7 UCD School of Biomolecular and Biomedical Science, University College Dublin, Dublin 04, Ireland; 8 Tipperary, Ireland; 9 UCD College of Social Sciences & Law, University College Dublin, Belfield, Dublin 4, Ireland

**Keywords:** Patient participation, Musculoskeletal diseases, Research, Ecosystem, Rheumatic diseases, Arthritis, Policy, Research priorities, Ownership, Public and patient involvement

## Abstract

**Background:**

Research priority setting is a useful approach to decide which unanswered questions are most worth trying to solve through research. The aim is to reduce bias in the research agenda. Traditionally, research was decided by funders, policymakers, and academics with limited influence from other stakeholders like people living with health conditions, caregivers, or the community. This can lead to research gaps that fail to address these important stakeholder needs. The objective of this study is to identify the top research priorities for Rheumatic and Musculoskeletal Disease (RMD) research in Ireland.

**Methods:**

The process framework included a design workshop, two online surveys and a review of the literature.

**Participants:**

545 people completed the first survey to identify RMD research topics relevant to Ireland, of which 72% identified as a person living with RMD. 460 people completed the second survey to prioritise these research topics.

**Results:**

The first survey had 2185 research topics submitted. These were analysed and grouped into 38 topic areas which were ranked in the second survey. The top three research priorities for RMD research in Ireland focused on preventing RMD progression, RMD diagnosis and its impact, and pain management.

**Conclusions:**

The prioritised research topics indicate important areas of RMD research for Ireland. Research funded in response to these co-created research priorities will have increased relevance and impact.

**Supplementary Information:**

The online version contains supplementary material available at 10.1186/s41927-022-00285-9.

## Key points

- What gets researched is typically decided by a top-down approach, which can lead to under-representation of research important to other stakeholders.
- This study uses a bottom-up approach to understand what rheumatic and musculoskeletal disease (RMD) research priorities are most important to multi-stakeholders in Ireland
- In addition to better diagnosis and treatment, we identified a number of traditionally under-researched areas as priorities for rheumatic and musculoskeletal research, including mental health, pain and diet.
- There is significant evidence uncertainty in the top 20 research priorities identified. This uncertainty exists both for an Irish context, but also more generally. This highlights the need to focus on underrepresented areas of research to improve the quality of life for people living with RMDs.

## Background

In Ireland, there has been a dedicated effort to engage a wider variety of stakeholders and the general public in all aspects of health research [1]. The Irish government releases national research priority areas. Recently, they have initiated a programme to incorporate public views on research [2]. There is increasing recognition of the historical bias in the research agenda and knock-on evidence disbalance [3]. These developments acknowledge gaps and uncertainty in the evidence base. Acknowledging this uncertainty, recognises that the judgement involved in assessing incomplete bodies of evidence are not solely dictated by scientific reasoning. Thus, the values and knowledge of “non-experts” can have a valid role in the decisions around research priorities.

Whereas national level prioritisation of research is useful in selecting general areas of focus nationally, it does not identify what research should be undertaken within these areas. Research in rheumatic and musculoskeletal disease (RMD) spans broad and diverse areas, with research priorities largely driven by individual researcher/research group interests and expertise. Research priorities are also driven by the research funding landscape, for example where a funding body launches a funding call targeted at a specific condition or topic. This traditional approach to the identification of research priorities and subsequently the research conducted may lack input from key stakeholders including people living with RMDs, carers, and healthcare professionals. Thus, in some cases the research priorities of people living with RMDs and other relevant stakeholders, have not been explored.

Seeking stakeholder perspectives is critical to informing research priorities to focus research resources in key areas. The involvement of public and patient involvement (PPI) partners can also inform such research priorities from a unique perspective. A comprehensive and cohesive research prioritisation programme has not been defined within Ireland beyond the governmental context. The current approach poses challenges given the competitive nature of attaining funding to support research projects. The lack of a strategic approach to the conduct of research in RMDs within the Ireland also acts as a barrier to engagement and collaboration between the RMD academic and research community.

Priority setting partnerships provide a valuable means of identifying research priorities. Such prioritisation can serve to aid organisations such as charitable groups work with funding agencies to develop themed calls linked to the priorities. The overall connectivity across the research-patient-health ecosystem possible through the research priority setting (RPS) approach provides confidence in the value of the research undertaken. The RPS priorities can result in new funding for research and also lead to new relationships developing that benefits the overall research ecosystem [4]. At a policy level, prioritisation exercises add value to the decision-making process by bridging the gap between the public/patient and the academic community leading to research that has greater overall societal impact.

Here, we present the process and results from a national RPS for arthritis and rheumatic disease research in Ireland. The project was undertaken by a multi-disciplinary team of researchers and people living with rheumatic diseases following a deliberative decision-making model. The output of this RPS is designed to facilitate more socially, economically, and clinically impactful decisions about arthritis and RMD research in Ireland.

## Methods

### Statement of ethics

This study was granted exemption from full ethical review by the University College Dublin Human Research Ethics Committee—Sciences (LS-E-20-38-Dorris). Anonymous surveys were used and no identifiable data was collected. Digital informed consent was obtained as part of the survey (Additional file 2).

### Public and patient involvement

PPI contributors were full partners in this study, involved from conception, throughout design and conduct of the study, and in the analysis, preparation and revision of the manuscript.

### Governance and team

The initiative was developed by one of the authors (ED) after consultation with peers, patient insight partners and Arthritis Ireland, Irelands national and largest charity dedicated to supporting people with arthritis, rheumatic diseases, and musculoskeletal disorders. The Arthritis Ireland Research Working Group acted as the governance committee. This consisted of two senior members of Arthritis Ireland, a patient member of Arthritis Ireland, the Chairs of Rheumatology at two major Irish Universities, consultant rheumatologists, a research scientist, and a professor in physiotherapy and vice president for research at an Irish University.

The project team consisted of a research scientist with experience in RPS. There were three patient insight partners, all of whom had previous PPI experience, and all of whom had personal experience in different RMD areas and were located in different geographical locations within Ireland. The remaining team comprised two academic physiotherapists: a postdoctoral researcher with experience in mixed methods and a professor of physiotherapy with significant experience in research policy.

### Process framework

Viergever et al.’s checklist for health research priority setting was used to plan the RPS process [5].

### Situational analysis and development of scope

The scope of the research prioritisation was defined via situational analysis of the Irish research context. This consisted of an analysis of peer reviewed and grey literature related to how research decisions are currently made in Ireland; who makes research decisions in Ireland; Who are the influential actors in research decision making; What policy and procedural documents are in place for decision making in RMD research in Ireland; and whether there is scope for improvement in research decision making practices. We used online search engines including PubMed and Google Scholar, organizational databases including Lenus Irish Health Repository and the Houses of the Oireachtas Library Digital Collections.

We analysed if there was a need for research priority setting for arthritis and rheumatic diseases in Ireland, and if so, what was the best way to approach it. This refined the prioritisation into five key areas: 1. The problem: research to measure the size of the health problem associated with RMDs in Ireland; 2. The cause: research to understand the causal agents and determinants of RMD and RMD-related health issues; 3. Solutions: research into what new interventions, tools, medicines, therapeutics are needed; 4. Policy & Practice: research to translate new interventions into policy and practice and understanding the barriers to delivering known interventions; 5. Health Impact: research to monitor and evaluate the effectiveness or health impact of an interventions or programmes.

### Design workshop

This phase was aimed at defining the selection and analysis criteria for RPS. In later steps we used surveys to gather information. We needed to know if (1) different stakeholders understood research prioritisation differently and (2) how to define if we had successfully captured enough information in these surveys.

We held a workshop with mixed stakeholder attendees including people living with rheumatic diseases, family members, junior doctors, consultant rheumatologists, allied health care professionals, researchers, and charity advocates.

The workshop used a banquet style layout with a facilitator and a note taker at each table. The tables consisted of mixed stakeholders. All facilitators were briefed in advance and had a facilitators guide to direct the workshop. The tables were covered in white paper tablecloths, which attendees were encouraged to write on should they wish to leave any additional thoughts or feedback.

The workshop used two main techniques: Mind Mapping and MoSCoW. Mind Mapping allowed attendees to deconstruct complex topics into a graphical representation of constituent subtopics and related themes. MoSCoW is an acronym derived from four prioritization categories (Must have, Should have, Could have, and Won't have). The workshop was designed to generate ideas using the mind map technique, and to use MoSCoW to narrow the scope. This combined method produced a strategy for the RPS, with all stakeholders agreeing on a single, clear set of deliverables for each stage of a project in a transparent way.

The design workshop facilitated better understanding of what was expected of the priority setting exercise from different stakeholder, which allowed better design of the survey to meet these expectations. The design workshop also informed the analysis phase. There were particular themes of research that the workshop anticipated should be reflected in the survey responses if the survey was conducted effectively and survey dissemination reached the intended diverse stakeholders. These acted as performance indicators for the survey. For example, under the key area “The Problem: Research to measure the size of the health problem in Ireland”; through the workshop we anticipated input on questions related not just to prevalence, but to a more diverse interpretation including social, psychological and economic costs of RMD. If we did not receive a diversity of interpretation in the survey data at the interim analysis, the design of the survey and its dissemination would be reviewed.

### Identification of research topics: survey design

A survey was designed for people to submit what topics/areas they thought needed to be researched to improve quality of life for people living with RMD in Ireland. The survey can be found in supporting materials. The survey was completed anonymously. It stated explicitly on the survey that “by participating in this survey you consent to the use of your anonymous data”.

The survey was reviewed and revised for clarity by a communications specialist, two people for whom English was not their first language, representatives of the key audience for the survey including people living with RMD (n = 4), family members (n = 2), clinicians (n = 3), allied health care professionals (n = 2), charity advocates (n = 2), and researchers (n = 2).

The survey was divided into two sections. Section one collected demographic information and section two collected the research questions the respondent would you like to see answered by RMD research. The survey collected the following demographic data on respondents: stakeholder category, age band, sex, ethnic/cultural background, province of Ireland lived in, and community.

There were five subsections in section two, and respondents could submit up to three questions in each subsection. The survey was only available in English. There was a mechanism to request a paper version of the survey and request assistance to help complete the survey via phone, email or post. The eSurvey was conducted using the Survey Monkey platform.

The survey was publicly available for anyone to complete. It was launched on the 06 April 2020 and open for six weeks. It was available on the Arthritis Ireland (Irish National RMD charity) website. It was sent to Irish professional organizations for rheumatologists, physiotherapists, nurses and other health care professionals. It was sent out to researchers investigating different aspects of rheumatic diseases in Universities across Ireland by email. Two reminder emails were sent, to encourage participation. The survey link was included in each email. The survey was also advertised on social media. No incentives were offered for completion. Unique survey respondents were measured via IP address. Cookies were not used.

### Survey analysis

An IP check was performed to identify potential duplicate entries. No duplicate entries were identified. All surveys, including incomplete surveys, were analysed.

There were 2185 research questions submitted by 545 respondents to the survey. Many people will ask a similar question in different ways. Thus, the submitted questions were analysed and grouped into themes.

#### Scope

Every submitted question was assessed to determine if it was within scope of the RSP. Respondents had been asked not to refer to specific RMDs in their responses. The responses should relate to arthritis and RMD research generally. If a respondent had simply answered with a condition that answer is out of scope.

- Real Examples:
- ❖ “Fibromyalgia”
- > Out of scope
- ❖ “Are symptoms attributed to fibromyalgia in people with Psoriatic Arthritis really a distinct entity or should they be considered part of the diagnosis of Psoriatic Arthritis?”
- > In scope. Classed as *Impact of co-morbidities on diagnosis*.

#### Data quality expectations

The submitted questions were compared with the criteria identified during the design workshop to determine if the expected diversity of submissions was achieved. The demographic data was also assessed to determine if a diverse group of respondents had been reached and if different communities, geographies and stakeholder types had been reached.

#### Grouping

The aim is to group similar questions together to identify the unique questions/areas. This was done by independent researchers, not in the field of rheumatic disease, to reduce potential bias in interpretation. All topics were analysed by two separate researchers, who had to agree that the questions, or topics, should be grouped together. Any disparity between the two researchers were brought to a third researcher and discussed for consensus. A member of the project team reviewed the research themes and analysis. The project them as a whole then reviewed and agreed upon all the thematic grouping of the submitted questions. This reduced the list down to thirty-eight major research themes.

### Research topic ranking (prioritisation) survey

A voluntary open survey hosted on the survey monkey platform was designed. This consisted of two pages. The first page gave information and instructions about the study. Page two consisted of two questions: Question one was “. Choose and rank your top ten most important research questions from the list below. With 1 being the most important, 2 the second most important and so on.” Question two was a free text space that asked, “Is there anything else you would like to tell us?”.

Two versions of the survey were used. Both were worded the same. However, feedback on the first survey, which used ranking dropdown as the answer method, was that it was cumbersome to complete on certain mobile devices. Thus, survey version two had a mobile-friendlier interface by using a matrix format, with the 38 questions to be ranked as row items and the ranking options of 1st Priority, 2nd Priority, 3rd Priority……., 10th Priority, NOT a top priority for me or N/A as the column choices. The matrix version, survey version two, did not automatically prevent more than one question being given a first priority rank.

The survey was publicly available for anyone to complete. It was launched on the 11 November 2020 and open for twelve weeks. It was disseminated via the same channels as the previous survey. No incentives were offered for completion. Unique survey respondents were measured via IP address. Cookies were not used. Demographic data was not collected for this survey.

### Analysis of the ranking survey

Each rank was given a weighted score.

**Table:** 

Priority rank	1st	2nd	3rd	4th	5th	6th	7th	8th	9th	10th
Weighted score	10	9	8	7	6	5	4	3	2	1

When data from survey version two was being recoded with the weighted scores, cases where more than one question had been given the same ranking were adjusted. For example, if a respondent gave three questions a first priority, each of these questions would be given a score of 8, and subsequent rankings adjusted (the second priority a score of 7, the third a score of 6 and so on). No one respondent could give a combined score of > 55 in total. This prevented skewing of the results by any one individual. The scores for each of the thirty-eight questions were summed to determine a ranking of the research topics by priority.

#### Internal validity check between survey versions

There was n = 272 respondents to survey version one and n = 192 respondents to survey version two. An IP check was performed to identify potential duplicate entries. No duplicate entries were identified. All surveys, including incomplete surveys, were analysed. Surveys were checked for duplicate entries. None were observed.

The rankings of each version were compared to each other and to the final/overall ranking. If the format did not make an impact, we would expect there to be similar rankings. There was good internal consistency between the versions. Overall, the consistency between versions was very strong, particularly within the top 10 (Additional file 1: Fig. S1).

### Top 20 identified research topics: analysis of the literature

**Fig. 1:**
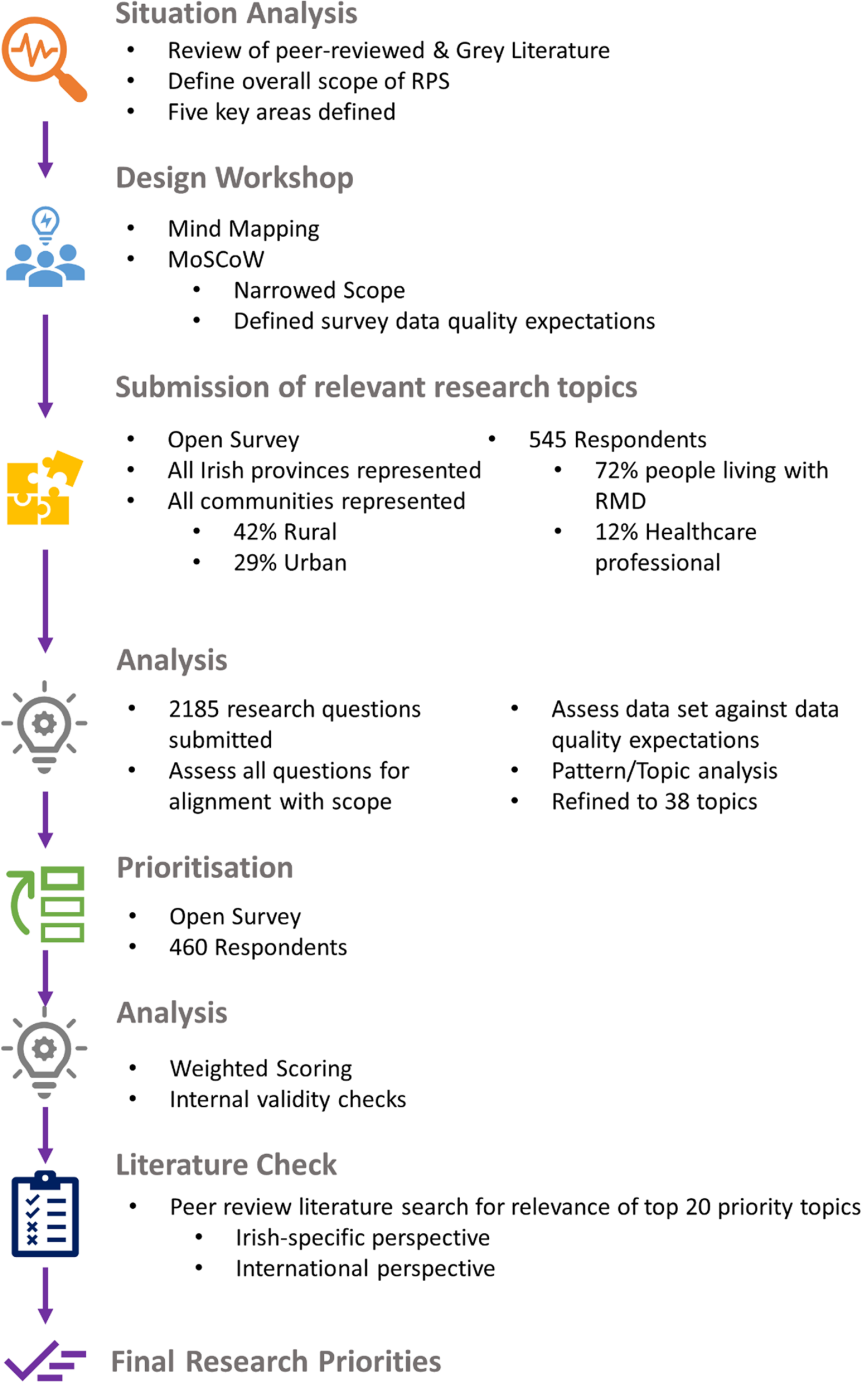
Overview of the RSP process. Image is original and created by the authors

A search of peer-reviewed original research, systematic review, and evidence synthesis literature was performed using online search engines including PubMed and Google Scholar. Grey literature was not included. Literature was searched for inclusion of Irish subjects or data within the study population. Most research is condition-specific, and therefore the search included articles related to “arthritis” “rheumatic disease” “RMD” “rheumatic and musculoskeletal Disease” and “rheumatology”. The search was not time-bound. Sample references are all within 15 years of the search. The aim was to identify recent literature (or lack thereof) to determine that the top 20 ranked research questions had indeed not yet been answered yet from 1. an Irish Perspective and 2. An international perspective. That could be a well cited, high impact publication in the field that says "more research is required" or a systematic review or similar (Fig. 1).

## Results

### Research topic submission survey

There were 545 respondents to the survey. There were 2185 topics submitted, of which 160 were determined to be out of scope. Demographic details of the respondents can be found in Additional file 1: Table S1. 22/545 (4%) of respondents provided partial demographic details and did not submit any research topics. One respondent did not submit any data. We had representation from the four provinces of Ireland (Leinster 48.07%, Munster 23.36%, Connacht 21.43%, and Ulster 5.02%). Compared to the population of the provinces, Connacht is overrepresented and Leinster is underrepresented in our dataset [6]. We had representation from all community types (urban (28.96%), suburban (27.41%), rural (42.28%) and island (< 1%)).

Men (n = 96) were relatively underrepresented in the sample compared to women (n = 416). The ethnic/cultural background of the sample was predominately white Irish (n = 479). The Irish population is also predominantly white Irish (82.2% 2016 census data [7]). However, compared to the proportional representation in the general population, Traveller and Asian communities are underrepresented in our data by approximately half, and Black/Black-Irish is underrepresented at approximately one quarter of what we would have expected to be reflective of the general population ([7], Additional file 1: Table S1). Interim analysis identified a total lack of representation from the Traveller community. In response, we proactively engaged with Pavee Point, the Traveller and Roma centre, to increase engagement.

The themes of research that the workshop anticipated should be reflected in the survey responses if the survey was conducted effectively and survey dissemination reached the intended diverse stakeholders were present in the data. Pattern and topic analysis grouped the submitted topics into 38 RMD research topics of priority for Ireland.

### Ranking survey

**Fig. 2:**
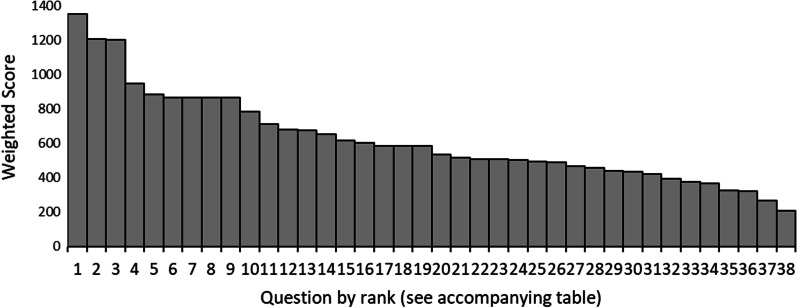
Distribution of weighted scores for ranked research priorities

There were 460 respondents to the ranking survey. The results of priority ranking and distribution of the weighted scores can be found in Fig. 2 and Table 1. The median weighted score for the 38 questions being prioritised is 560 and the mean score is 619.66 (95% CI: 536.92–702.40). The top 10 ranked research priorities have scores ranging from 1352–785, with priorities ranked 11–20 ranging from 709–584, and remaining topics scoring between 508–207.

**Table 1:** Ranked research priorities

Rank	Question	Score
1	How can we best prevent or reduce the progression/worsening of disease in people living with arthritis and rheumatic diseases?	1352
2	Can we improve early diagnosis of arthritis and rheumatic diseases? What impact would earlier diagnosis have on quality of life?	1207
3	How can pain be better managed for those living with arthritis and rheumatic diseases? What factors influence access to adequate pain relief and pain management?	1199
4	Can we develop or improve medications to treat rheumatic diseases?	945
5	What impact does exercise have on the risk, prevention or management of arthritis and rheumatic diseases?	881
6	Can we improve our knowledge of the biology of rheumatic diseases to make better treatments or treatment choices?	866
7	What impact does diet have in the management of rheumatic diseases?	866
8	How can we improve the diagnosis of arthritis and rheumatic diseases?	865
9	What impact does arthritis and rheumatic disease have on mental health? How can this best be treated, managed or improved?	864
10	What role does genetics play in the risk of having a rheumatic disease? Can genetics help to guide treatment or management strategies?	785
11	What influences speed of diagnosis, access to consultants and treatment decisions in Ireland? Does it vary by geography, socioeconomic factors or hospital group?	709
12	What role does non-pharmaceutical interventions, such as lifestyle changes, have in disease management?	681
13	Can we improve self-management and disease monitoring? Does improved disease monitoring or investment in self-management strategies lead to improved outcomes and quality of life for people living with rheumatic diseases?	676
14	Would electronic health records improve delivery of healthcare and disease management? Would patient access to their own electronic health records improve informed choice and doctor-patient partnerships in disease management?	650
15	Do patients receive clear communication in relation to the treatment and or management of disease? How might this be improved?	618
16	Is there a relationship between rheumatic disease and female sex hormones, fertility, menstruation or pregnancy?	603
17	Does surgery or joint replacement lead to improved patient outcomes and quality of life?	586
18	What is the financial burden associated with rheumatic disease and self-management in Ireland?	586
19	What is the impact of arthritis and rheumatic diseases on employment, careers and schooling in Ireland?	584
20	Can national government policy improve outcomes and quality of life for people living with rheumatic diseases in Ireland? What can Irish policy makers learn from other countries to improve policy in Ireland?	536
21	What environmental factors have a role to play in arthritis and rheumatic diseases? Can we use this knowledge to create a healthier community or environment to live in?	518
22	Does access to health services and community support depend on where you live? If so, what is the impact of this?	508
23	What role do lifestyle factors play in the risk of developing a rheumatic disease?	506
24	What is the impact of comorbidities (other co-occurring medical conditions) in rheumatic diseases? Do different treatment strategies affect the risk of comorbidities?	503
25	Is there adequate access to relevant, reliable information, help and support for people living with a rheumatic disease?	492
26	What impact does involving people living with rheumatic diseases in healthcare policy, decisions and research have?	487
27	How does Ireland compare to other EU countries in terms of treatment, outcomes and quality of life for people living with arthritis and rheumatic disease? Can we use this knowledge to improve quality of life for people living with rheumatic diseases in Ireland?	468
28	What factors influence patient-access to medications and non-pharmaceutical treatments?	456
29	Would better public awareness of rheumatic diseases improve early diagnosis or disease prevention?	439
30	How does Ireland compare to other EU countries in the management and treatment of arthritis and rheumatic diseases? How does Irish policy, healthcare spending, resources and staffing related to arthritis and rheumatic diseases compare to other European countries?	436
31	How can arthritis and rheumatic disease be better classified? Does better classification improve disease outcome and or prognosis?	422
32	How common is arthritis and rheumatic diseases in Ireland? Is there any association between where you live and risk of being diagnosed with a rheumatic disease?	393
33	Are there adequate supports for treatment and self-management of arthritis and rheumatic diseases in those with intellectual disabilities?	374
34	What is the impact of education on disease management?	367
35	Does sex or gender have an impact on the choice of effective treatment strategies? Is there a difference in disease response between the sexes?	325
36	How does Covid-19 impact arthritis and rheumatic disease management in Ireland? Are these impacts similar to that of other European countries?	321
37	What is the socioeconomic impact of rheumatic diseases in Ireland?	266
38	Does familial support lead to improved disease outcomes and quality of life?	207

### Evidence uncertainty in top ranked research priorities

A literature search of the top ranked research priorities demonstrated there was evidence uncertainty for all top priorities (Additional file 1: Table S2). Of the top 20 priorities, 15 had either no studies or only small-scale studies published from an Irish-specific perspective. Further literature was available from an international perspective, however, literature relating to the impact of national government policy, the impact of RMD on employment, careers, and schooling and the impact of clear communication about treatment and management tended to be regionally restricted or largely underrepresented in more recent literature.

## Discussion

Here we identified the top research priorities for arthritis and RMD research in Ireland. Of note is the scope of the priorities identified. In addition to clinical and health services-related priorities, there was focus on self-management factors, such as diet and exercise, and secondary impacts such as pain and mental health.

The identification of RMD research priorities presents an excellent opportunity for a more cohesive and focussed research strategy within the Irish context. Stakeholders now have a definitive list of research topics which are deemed of significant importance to the RMD community. This should guide the research strategy of research groups, harnessing the expertise of the academic and PPI research community to examine specific topics. This targeted approach can facilitate increased collaboration and lead to enhanced research outputs and impacts to the key areas identified in this prioritisation. A focussed approach to research has the potential to impact beyond research, by highlighting key issues and increasing the depth and breadth of research on prioritised topics [8]. There will be scope to influence healthcare policy and delivery with a more robust body of research evidence, thereby enhancing the health outcomes of people with RMDs.

Health research charities provide significant funding for research [9]. The identification of national research priorities can help to guide charities in the direction of what research to fund. It can also be very useful in the development of research strategies and fundraising as it gives a truer sense of the needs and challenges of the community as a whole [10]. The priorities identified here have been used by the national Irish RMD charity Arthritis Ireland in their latest funding call [11]. They are also being used to inform their research strategy. Having an evidence-based, transparent set of priorities identified by the community the charity represents can greatly assist in communicating clear needs and goals to potential donors.

RMDs are complex conditions with a wide impact on peoples’ lives. There are no easy answers when it comes to understanding their related impact on social issues, healthcare, and quality of life. While this RSP was undertaken in Ireland, given the evidence uncertainty present for the top 20 priorities it is reasonable that the findings of this study are relevant to groups in other countries considering undertaking an RSP.

## Study limitations

This study aimed to include a broad range of different stakeholder experiences. Whereas the geographic and community type engaged was diverse, the study population was not wholly reflective of the Irish population. In particular, although we engaged some minority groups, the relative engagement of Traveller and Black/Black-Irish ethnicities was suboptimal. We developed an approach to RPS to meet our specific needs. This methodology was informed by both the James Lind Alliance (JLA) [12] and the World Health Organisation’s priority setting methods [13, 14]. RMDs impact all aspects of life and we did not want to specifically focus on clinical or health systems research. As such the JLA approach of only including patients, carers and clinicians was deemed to narrow. Thus, rather than following the well characterised JLA approach, a bespoke process framework was developed.

In an effort to be non-prescriptive in the relevant research questions for prioritisation, we used an open survey to invite people to submit the questions or topic relevant to them. Asking respondents to identify a research question, rather than presenting a list of predetermined questions, requires a higher degree of health literacy from respondents. We actively tried to minimise this by including explainers in the instructions for the survey, having the language of the survey reviewed by a number of different people with different levels of experience, using manual rather than automated coding to account for a variety of language used and diversity in language expression by individuals, and offering the opportunity to have the survey in an alternative format. However, it is likely that even with these additional considerations, the open approach is likely to have excluded certain people. We cannot exclude potential health literacy impacts of this research design.

## Conclusion

Research funded in response to these co-created research priorities should have increased relevance and impact. As the priorities were developed together with all stakeholders who have an interest in improving the quality of life for people living with RMDs, there is shared ownership. This provides a strong foundation to continue the process of collaboration between people living with RMDs, advocating for RMDs, researchers and healthcare professionals.

## Supplementary Information

**Additional file 1. Supplementary Figure 1.** Internal validity between ranking eSurvey versions. **Supplementary Table 1. **Demographic details of respondents to the research topic submission survey. **Supplementary Table 2.** Evidence uncertainty in top ranked research priorities.

**Additional file 2.** Research Topic Submission Survey.

**Additional file 3.** Plain language summary.

## Data Availability

Raw survey data available upon reasonable request from the corresponding author.

## Abbreviations

CI: Confidence interval
PPI: Public and patient involvement
RMD: Rheumatic and musculoskeletal disease
RPS: Research priority setting
