# Supplementary material for: Priorities for rheumatic and musculoskeletal disease research in Ireland
Source: BMC Rheumatol. 2022 Aug 11;6:55. doi: 10.1186/s41927-022-00285-9 (PMC9365446; doi:10.1186/s41927-022-00285-9)
Supplement: Supplementary file 1 — Additional file 1. Supplementary Figure 1. Internal validity between ranking eSurvey versions. Supplementary Table 1. Demographic details of respondents to the research topic submission survey. Supplementary Table 2. Evidence uncertainty in top ranked research priorities. [file 41927_2022_285_MOESM1_ESM.docx]

Supplementary figure 1: Internal validity between ranking eSurvey versions.

The ranking of each survey version was compared to each other and to the final ranking. The ranking of respondents that used survey version 1 (V1, orange square) and survey version 2 (V2, grey diamond) is shown overlaid onto the total (final, blue circle) research question ranking. If the format did not make an impact, we would expect there to be similar rankings. There was good internal consistency between the versions. Of the final top 10 priorities (circle, blue), 9 were found in each survey version’s top 10. Overall, the consistency between versions is very strong, particularly within the top 10.

Supplementary Table 1: Demographic details of respondents to the research topic submission survey

|  | | | **%** | **n** |
| --- | --- | --- | --- | --- |
| **Stakeholder Type** | | | | |
| Person with a Rheumatic Disease | | | 71.64% | 384 |
| Healthcare professional | | | 11.94% | 64 |
| Family member of someone with a Rheumatic Disease | | | 6.72% | 36 |
| Researcher | | | 2.99% | 16 |
| Carer/former carer of someone with a Rheumatic Disease | | | 1.12% | 6 |
| Friend of someone with a Rheumatic Disease | | | 0.93% | 5 |
| Social care professional | | | 0.19% | 1 |
| Other | | | 4.48% | 24 |
|  | | |  |  |
|  | | | *Skipped* | *8* |
| **Sex** | | |  |  |
| Male | | | 17.74% | 96 |
| Female | | | 76.90% | 416 |
| Other | | | 0.18% | 1 |
| Prefer not to say | | | 1.11% | 6 |
|  | | |  |  |
|  | | | *Skipped* | *22* |
| **Age** | | |  |  |
| >25 | | | 2.40% | 13 |
| 25-44 | | | 35.67% | 193 |
| 45-64 | | | 49.17% | 266 |
| >65 | | | 12.75% | 69 |
|  | | |  |  |
|  | | | *Skipped* | *0* |
| **Geographic Location** | | | | |
| Leinster | | | 48.07% | 249 |
| Munster | | | 23.36% | 121 |
| Connacht | | | 21.43% | 111 |
| Ulster | | | 5.02% | 26 |
| Other | | | 2.12% | 11 |
|  | | |  |  |
|  | | | *Skipped* | *26* |
| **Community Type** | | |  |  |
| Rural | | | 42.28% | 219 |
| Urban | | | 28.96% | 150 |
| Suburban | | | 27.41% | 142 |
| Island | | | 0.19% | 1 |
| Other | | | 1.16% | 6 |
|  | | |  |  |
|  | | | *Skipped* | *26* |
| **Ethnic/Cultural background** | | |  |  |
| White | | | 93.16% | 504 |
|  | White Irish | 479 |  |  |
|  | White Traveller | 2 |  |  |
|  | White: any other white background | 23 |  |  |
| Black/Black Irish | | | 0.37% | 2 |
|  | African | 1 |  |  |
|  | Any other black background | 1 |  |  |
| Asian/Asian Irish | | | 0.74% | 4 |
|  | Chinese | 0 |  |  |
|  | Any other Asian Background | 4 |  |  |
| Other, including mixed background | | | 0.37% | 2 |
| Prefer not to say | | | 0.92% | 5 |
|  | | |  |  |
|  | | | *Skipped* | *24* |

Supplementary Table 2: Evidence uncertainty in top ranked research priorities

| **Rank** | **Question** | **Irish Perspective** | **Sample Reference**  **[Irish Context]** | **Intl. Perspective** | **Sample Reference**  **[International Context]** |
| --- | --- | --- | --- | --- | --- |
| 1 | How can we best prevent or reduce the progression/worsening of disease in people living with arthritis and rheumatic diseases? | -RMD-specific  -Not exhaustive | (Corciulo, Lendhey et al. 2017) | -RMD-specific  -Region-specific  -Not exhaustive | (Wang, Sampson et al. 2013) |
| 2 | Can we improve early diagnosis of arthritis and rheumatic diseases? What impact would earlier diagnosis have on quality of life? | -RMD-specific  -Not exhaustive  -Less evidence on impact | (Haroon, O'Rourke et al. 2015, FitzGerald and Pennington 2022) | -RMD-specific  -Region-specific  -Not exhaustive  -Less focus on impact and Quality of Life | (Finckh, Liang et al. 2006) |
| 3 | How can pain be better managed for those living with arthritis and rheumatic diseases? What factors influence access to adequate pain relief and pain management? | -RMD-specific  -Not exhaustive  -Very little research literature on access | (Giggins, Fullen et al. 2012, Dunlevy, MacLellan et al. 2019) | -RMD-specific  -Region-specific  -Not exhaustive  -Less focus on access | (Woolf, Carr et al. 2008, Geenen, Overman et al. 2018) |
| 4 | Can we develop or improve medications to treat rheumatic diseases? | -RMD-specific  -Not exhaustive  -Little evidence from Irish-designed/innovated medications | (Coll, Robertson et al. 2015, Gao, McGarry et al. 2016) | -RMD-specific  -Not exhaustive | (Deodhar, Mease et al. 2019, Xie, Huang et al. 2019) |
| 5 | What impact does exercise have on the risk, prevention or management of arthritis and rheumatic diseases? | Only small scale Irish interventations or research studies identified | n/a | -RMD-specific  - Region-specific  -Not exhaustive | (Rongen-van Dartel, Repping-Wuts et al. 2015, Grønne, Roos et al. 2021) |
| 6 | Can we improve our knowledge of the biology of rheumatic diseases to make better treatments or treatment choices? | -RMD-specific  -Not exhaustive  -Most evidence is in the autoimmune & inflammatory arthritis area | (Dorris, Tazzyman et al. 2019) | -RMD-specific  - Region-specific  -Not exhaustive | (Martinon, Pétrilli et al. 2006) |
| 7 | What impact does diet have in the management of rheumatic diseases? | No powered Irish interventations or research studies identified | n/a | -RMD-specific  - Region-specific  -Few powered studies  -Not exhaustive | (Zhang, Chen et al. 2012, Thomas, Browne et al. 2018) |
| 8 | How can we improve the diagnosis of arthritis and rheumatic diseases? | -Largely restricted to a few RMDs (Psoriatic Arthritis and Rheumatoid Arthritis)  -Few using Irish cohorts | (Mc Ardle, Flatley et al. 2015) | -RMD-specific  - Region-specific  -Not exhaustive | (Descamps, Olagne et al. 2018) |
| 9 | What impact does arthritis and rheumatic disease have on mental health? How can this best be treated, managed, or improved? | Only small, localised studies identified in Irish populations | n/a | -RMD-specific  - Region-specific  -Not exhaustive  - focus on association studies  - lacking impact studies | (Matcham, Rayner et al. 2013, Chang, Hsu et al. 2015) |
| 10 | What role does genetics play in the risk of having a rheumatic disease? Can genetics help to guide treatment or management strategies? | -RMD-specific  -Not exhaustive  - Lack of population-level studies  -Most studies are rare disease populations, or case-specific | (Adeeb, Dorris et al. 2021, O'Leary, Killeen et al. 2021) | -RMD-specific  - Region-specific  -evidence of both genetic risk and treatment association studies  -Not exhaustive | (Stahl, Raychaudhuri et al. 2010, Eyre, Bowes et al. 2012) |

Adeeb, F., E. R. Dorris, N. E. Morgan, D. Lawless, A. Maqsood, W. L. Ng, O. Killeen, E. P. Cummins, C. T. Taylor, S. Savic, A. G. Wilson and A. Fraser (2021). "A Novel RELA Truncating Mutation in a Familial Behçet's Disease-like Mucocutaneous Ulcerative Condition." Arthritis Rheumatol **73**(3): 490-497.

Chang, M.-H., J.-W. Hsu, K.-L. Huang, T.-P. Su, Y.-M. Bai, C.-T. Li, A. C. Yang, W.-H. Chang, T.-J. Chen, S.-J. Tsai and M.-H. Chen (2015). "Bidirectional Association Between Depression and Fibromyalgia Syndrome: A Nationwide Longitudinal Study." The Journal of Pain **16**(9): 895-902.

Coll, R. C., A. A. Robertson, J. J. Chae, S. C. Higgins, R. Muñoz-Planillo, M. C. Inserra, I. Vetter, L. S. Dungan, B. G. Monks, A. Stutz, D. E. Croker, M. S. Butler, M. Haneklaus, C. E. Sutton, G. Núñez, E. Latz, D. L. Kastner, K. H. Mills, S. L. Masters, K. Schroder, M. A. Cooper and L. A. O'Neill (2015). "A small-molecule inhibitor of the NLRP3 inflammasome for the treatment of inflammatory diseases." Nat Med **21**(3): 248-255.

Corciulo, C., M. Lendhey, T. Wilder, H. Schoen, A. S. Cornelissen, G. Chang, O. D. Kennedy and B. N. Cronstein (2017). "Endogenous adenosine maintains cartilage homeostasis and exogenous adenosine inhibits osteoarthritis progression." Nat Commun **8**: 15019.

Deodhar, A., P. J. Mease, I. B. McInnes, X. Baraliakos, K. Reich, A. Blauvelt, C. Leonardi, B. Porter, A. Das Gupta, A. Widmer, L. Pricop and T. Fox (2019). "Long-term safety of secukinumab in patients with moderate-to-severe plaque psoriasis, psoriatic arthritis, and ankylosing spondylitis: integrated pooled clinical trial and post-marketing surveillance data." Arthritis Res Ther **21**(1): 111.

Descamps, L., L. Olagne, C. Merlin, F. Cachin, M. Soubrier and S. Mathieu (2018). "Utility of PET/CT in the diagnosis of inflammatory rheumatic diseases: a systematic review and meta-analysis." Annals of the Rheumatic Diseases **77**(11): e81.

Dorris, E. R., S. J. Tazzyman, J. Moylett, N. Ramamoorthi, J. Hackney, M. Townsend, M. Muthana, M. J. Lewis, C. Pitzalis and A. G. Wilson (2019). "The Autoimmune Susceptibility Gene C5orf30 Regulates Macrophage-Mediated Resolution of Inflammation." J Immunol **202**(4): 1069-1078.

Dunlevy, C., G. A. MacLellan, E. O'Malley, C. Blake, C. Breen, K. Gaynor, N. Wallace, R. Yoder, D. Casey, J. Mehegan, B. M. Fullen and D. O'Shea (2019). "Does changing weight change pain? Retrospective data analysis from a national multidisciplinary weight management service." Eur J Pain **23**(8): 1403-1415.

Eyre, S., J. Bowes, D. Diogo, A. Lee, A. Barton, P. Martin, A. Zhernakova, E. Stahl, S. Viatte, K. McAllister, C. I. Amos, L. Padyukov, R. E. Toes, T. W. Huizinga, C. Wijmenga, G. Trynka, L. Franke, H. J. Westra, L. Alfredsson, X. Hu, C. Sandor, P. I. de Bakker, S. Davila, C. C. Khor, K. K. Heng, R. Andrews, S. Edkins, S. E. Hunt, C. Langford, D. Symmons, P. Concannon, S. Onengut-Gumuscu, S. S. Rich, P. Deloukas, M. A. Gonzalez-Gay, L. Rodriguez-Rodriguez, L. Ärlsetig, J. Martin, S. Rantapää-Dahlqvist, R. M. Plenge, S. Raychaudhuri, L. Klareskog, P. K. Gregersen and J. Worthington (2012). "High-density genetic mapping identifies new susceptibility loci for rheumatoid arthritis." Nat Genet **44**(12): 1336-1340.

Finckh, A., M. H. Liang, C. M. van Herckenrode and P. de Pablo (2006). "Long-term impact of early treatment on radiographic progression in rheumatoid arthritis: A meta-analysis." Arthritis Rheum **55**(6): 864-872.

FitzGerald, O. and S. R. Pennington (2022). "HIPPOCRATES: improving diagnosis and outcomes in psoriatic arthritis." Nature Reviews Rheumatology.

Gao, W., T. McGarry, C. Orr, J. McCormick, D. J. Veale and U. Fearon (2016). "Tofacitinib regulates synovial inflammation in psoriatic arthritis, inhibiting STAT activation and induction of negative feedback inhibitors." Ann Rheum Dis **75**(1): 311-315.

Geenen, R., C. L. Overman, R. Christensen, P. Åsenlöf, S. Capela, K. L. Huisinga, M. E. P. Husebø, A. J. A. Köke, Z. Paskins, I. A. Pitsillidou, C. Savel, J. Austin, A. L. Hassett, G. Severijns, M. Stoffer-Marx, J. W. S. Vlaeyen, C. Fernández-de-Las-Peñas, S. J. Ryan and S. Bergman (2018). "EULAR recommendations for the health professional's approach to pain management in inflammatory arthritis and osteoarthritis." Ann Rheum Dis **77**(6): 797-807.

Giggins, O., B. Fullen and G. Coughlan (2012). "Neuromuscular electrical stimulation in the treatment of knee osteoarthritis: a systematic review and meta-analysis." Clin Rehabil **26**(10): 867-881.

Grønne, D. T., E. M. Roos, R. Ibsen, J. Kjellberg and S. T. Skou (2021). "Cost-effectiveness of an 8-week supervised education and exercise therapy programme for knee and hip osteoarthritis: a pre-post analysis of 16 255 patients participating in Good Life with osteoArthritis in Denmark (GLA:D)." BMJ Open **11**(12): e049541.

Haroon, M., M. O'Rourke, P. Ramasamy, C. C. Murphy and O. FitzGerald (2015). "A novel evidence-based detection of undiagnosed spondyloarthritis in patients presenting with acute anterior uveitis: the DUET (Dublin Uveitis Evaluation Tool)." Ann Rheum Dis **74**(11): 1990-1995.

Martinon, F., V. Pétrilli, A. Mayor, A. Tardivel and J. Tschopp (2006). "Gout-associated uric acid crystals activate the NALP3 inflammasome." Nature **440**(7081): 237-241.

Matcham, F., L. Rayner, S. Steer and M. Hotopf (2013). "The prevalence of depression in rheumatoid arthritis: a systematic review and meta-analysis." Rheumatology (Oxford) **52**(12): 2136-2148.

Mc Ardle, A., B. Flatley, S. R. Pennington and O. FitzGerald (2015). "Early biomarkers of joint damage in rheumatoid and psoriatic arthritis." Arthritis Research & Therapy **17**(1): 141.

O'Leary, D., O. G. Killeen and A. G. Wilson (2021). "Genetics of chronic nonbacterial osteomyelitis in the irish population: no significant association with rare FBLIM1 variants." Pediatr Rheumatol Online J **19**(1): 32.

Rongen-van Dartel, S. A., H. Repping-Wuts, M. Flendrie, G. Bleijenberg, G. S. Metsios, W. B. van den Hout, C. H. van den Ende, G. Neuberger, A. Reid, P. L. van Riel and J. Fransen (2015). "Effect of Aerobic Exercise Training on Fatigue in Rheumatoid Arthritis: A Meta-Analysis." Arthritis Care Res (Hoboken) **67**(8): 1054-1062.

Stahl, E. A., S. Raychaudhuri, E. F. Remmers, G. Xie, S. Eyre, B. P. Thomson, Y. Li, F. A. Kurreeman, A. Zhernakova, A. Hinks, C. Guiducci, R. Chen, L. Alfredsson, C. I. Amos, K. G. Ardlie, A. Barton, J. Bowes, E. Brouwer, N. P. Burtt, J. J. Catanese, J. Coblyn, M. J. Coenen, K. H. Costenbader, L. A. Criswell, J. B. Crusius, J. Cui, P. I. de Bakker, P. L. De Jager, B. Ding, P. Emery, E. Flynn, P. Harrison, L. J. Hocking, T. W. Huizinga, D. L. Kastner, X. Ke, A. T. Lee, X. Liu, P. Martin, A. W. Morgan, L. Padyukov, M. D. Posthumus, T. R. Radstake, D. M. Reid, M. Seielstad, M. F. Seldin, N. A. Shadick, S. Steer, P. P. Tak, W. Thomson, A. H. van der Helm-van Mil, I. E. van der Horst-Bruinsma, C. E. van der Schoot, P. L. van Riel, M. E. Weinblatt, A. G. Wilson, G. J. Wolbink, B. P. Wordsworth, C. Wijmenga, E. W. Karlson, R. E. Toes, N. de Vries, A. B. Begovich, J. Worthington, K. A. Siminovitch, P. K. Gregersen, L. Klareskog and R. M. Plenge (2010). "Genome-wide association study meta-analysis identifies seven new rheumatoid arthritis risk loci." Nat Genet **42**(6): 508-514.

Thomas, S., H. Browne, A. Mobasheri and M. P. Rayman (2018). "What is the evidence for a role for diet and nutrition in osteoarthritis?" Rheumatology (Oxford) **57**(suppl_4): iv61-iv74.

Wang, M., E. R. Sampson, H. Jin, J. Li, Q. H. Ke, H. J. Im and D. Chen (2013). "MMP13 is a critical target gene during the progression of osteoarthritis." Arthritis Res Ther **15**(1): R5.

Woolf, A., A. Carr, J. Frolich, M. Guslandi, B. Michel and H. Zeidler (2008). "Investigating the barriers to effective management of musculoskeletal pain: an international survey." Clin Rheumatol **27**(12): 1535-1542.

Xie, W., Y. Huang, S. Xiao, X. Sun, Y. Fan and Z. Zhang (2019). "Impact of Janus kinase inhibitors on risk of cardiovascular events in patients with rheumatoid arthritis: systematic review and meta-analysis of randomised controlled trials." Ann Rheum Dis **78**(8): 1048-1054.

Zhang, Y., C. Chen, H. Choi, C. Chaisson, D. Hunter, J. Niu and T. Neogi (2012). "Purine-rich foods intake and recurrent gout attacks." Ann Rheum Dis **71**(9): 1448-1453.
