# Supplementary material for: Priorities for rheumatic and musculoskeletal disease research in Ireland
Source: BMC Rheumatol. 2022 Aug 11;6:55. doi: 10.1186/s41927-022-00285-9 (PMC9365446; doi:10.1186/s41927-022-00285-9)
Supplement: Supplementary file 2 — Additional file 2. Research Topic Submission Survey. [file 41927_2022_285_MOESM2_ESM.pdf]

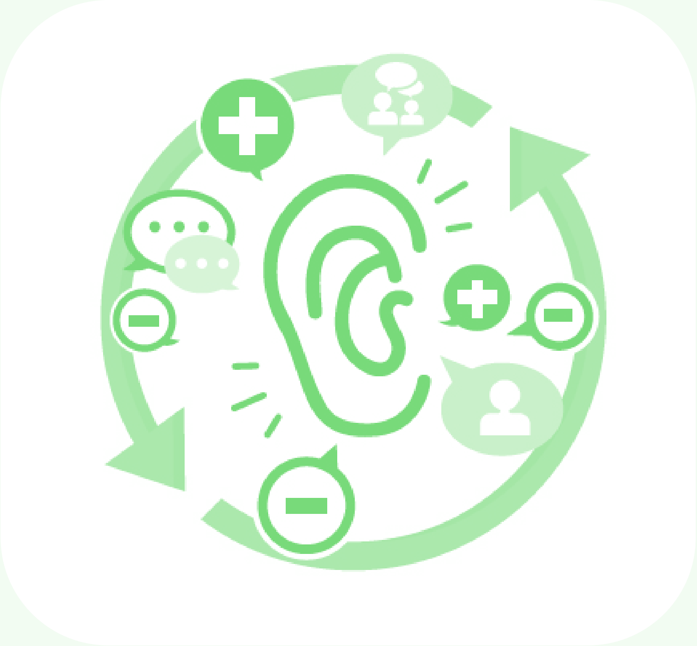

TELL US

What questions would you like to see answered by Arthritis and Rheumatic Disease research?

UCD CENTRE FOR ARTHRITIS RESEARCH

+353 1 716 6728

rheumatology@ucd.ie

Conway Institute | UCD Belfield | Dublin | D04 C7X2 | Ireland

IN PARTNERSHIP WITH

UCD DUBLIN

Patient Voice in Arthritis Research

UNIVERSITY OF LIMERICK
OLLSCOIL LUIMNIGH

Arthritis Ireland
Little Things *make a* Big Difference

We're asking people living with arthritis and rheumatic disease, health and social care professionals, and research professionals to **help us identify the top 10 unanswered questions they want research in Ireland to address.** This will help to guide research and enable researchers to focus on the needs of people living with the condition.

What will happen with my information?

The unanswered questions will be published but not linked to you. All personal data will remain confidential.

We do not collect identifiable information from you.

By participating in this survey you consent to the use of your anonymous data.

Who can take part?

People living with arthritis and rheumatic diseases, their friends, family and carers. Health and social care professionals, researchers and the interested public.

What do we mean by Arthritis and Rheumatic Diseases?

Rheumatic diseases are also called musculoskeletal diseases. There are over 200 different rheumatic diseases. Rheumatic diseases typically have an element of pain and a reduction in the range of motion and function of the joints but can also affect internal organs. In some rheumatic diseases there are signs of inflammation such as swelling, redness, warmth in the affected areas. Some people use the word arthritis to refer to all rheumatic diseases. Arthritis, which literally means joint inflammation, is just part of the rheumatic diseases. You can have a rheumatic disease at any age, including childhood. We want to hear from people living with any form of rheumatic disease, from Rheumatoid Arthritis to Connective Tissue Diseases; Spondyloarthropathies to Fibromyalgia; Gout to Juvenile Idiopathic Arthritis and all other types, be they rare diseases such as Behcets Syndrome or more common such as osteoarthritis.

What do we mean by unanswered questions?

We're looking for questions that, if fully answered by research, could make a real difference to people living with rheumatic diseases.

Do I have to check if the research question is unanswered?

No. You can write a short sentence about something that is important to you, but for

which you haven't been able to find an answer. The project team will then check against existing research to find out whether they have been answered or not.

Does it have to be a question?

No. You can write a short statement and the team collecting your responses will turn them into questions.

Do I have to answer every category?

No. The important thing is to draw on your personal experience of rheumatic disease and answer the categories that are important to you.

Why do you ask for questions about my background?

We want to know how representative the people who answered the questions are compared to the Irish overall population. This helps us to know how reflective the answers are of the overall community.

What will we do with the results?

We will use the results of this survey to guide future research in Ireland.

By sharing what we find with the international research community, we hope to refocus research on the needs of the rheumatic disease community as a whole.

Section 1

Q 1. Which of the following best describes you?

Person with a Rheumatic Disease □

Carer/former carer of someone with a Rheumatic Disease □

Family member of someone with a Rheumatic Disease □

Friend of someone with a Rheumatic Disease □

Health care professional □

Social care professional □

Researcher □

Other □

Q 2. What age are you?

Under 18 □

18-24 □

25-34 □

35-44 □

45-54 □

55-64 □

65-74 □

75-84 □

Over 84 □

Q 3. What is your sex?

Male □ Female □ Other □ Prefer not to say □

Q 4. What is your ethnic or cultural background?

A. White

Irish ☐

Irish Traveller ☐

Any other White background ☐

B. Black/Black Irish

African ☐

Any other Black background ☐

C. *Asian/Asian Irish*

Chinese ☐

Any other Asian background ☐

D. *Other,* including mixed background ☐

____

____

E. Prefer not to say ☐

Q 5. What province do you live in?

Connaught ☐

Leinster ☐

Munster ☐

Ulster ☐

Other ☐

Q 6. What type of area do you live in?

Urban ☐

Suburban ☐

Rural ☐

Islands ☐

Other ☐

Health and social care professionals only. What is your main profession?

____

____

Would you like to keep up-to-date with the next steps?

Find more information

www.ucd.ie/car/

and

www.arthritisireland.ie

1. The Issue

1. What question (s) about understanding the size and impact of arthritis and rheumatic disease would you like to see answered by research?

1. ____

2. ____

3. ____

2. The Cause

2. What question(s) about how and why people get arthritis would you like to see answered by research?

1. ____

2. ____

3. ____

3. The Solution

1. What question(s) about how to improve diagnosis, treatment and quality of life for arthritis and rheumatic disease would you like to see answered by research?

1. ____

2. ____

3. ____

4. Policy & Practice

2. What question(s) about how to improve the management of arthritis and rheumatic diseases would you like to see answered by research?

1. ____

2. ____

3. ____

5. Health Impact

1. What question(s) about how to monitor the long term impact of practice and interventions would you like to see answered by research?

1. ____

2. ____

3. ____
