# Supplementary material for: Priorities for rheumatic and musculoskeletal disease research in Ireland
Source: BMC Rheumatol. 2022 Aug 11;6:55. doi: 10.1186/s41927-022-00285-9 (PMC9365446; doi:10.1186/s41927-022-00285-9)
Supplement: Supplementary file 3 — Additional file 3. Plain language summary. [file 41927_2022_285_MOESM3_ESM.docx]

**Background to the research**

Research priority setting is a collective activity for deciding what questions or topics are most worth trying to solve through research. Traditionally, the health research agenda has been largely researcher- and politics-driven. There has been limited influence from people living with rheumatic and musculoskeletal diseases (RMDs), caregivers or the community. This can lead to research gaps that fail to address important needs of these stakeholders.

**People with RMDs and researchers worked together**

In our research, we identified what the top research priorities for RMD research in Ireland are. We formed a team of people living with RMDs, researchers, and healthcare professionals to work together. The core project team was formed from different parts of Ireland. It had six people; half were people living with RMDs and half were research academics. There was equal decision-making power and responsibilities for all members of the project team.

**How the research was carried out**

The team used a systematic approach to carry out the research priority setting. This means they looked at the best methods used by other teams or for other disease areas and adapted them to create a plan of action. The research approach used two surveys. The first survey asked people to tell us what topics they thought needed to be researched in order to improve the quality of life for people living with RMDs in Ireland. We received replies from 545 people who submitted more than 2000 research topics. Of the 545 people, 72% identified as a person living with RMD.

Many people will ask a similar question in different ways. Therefore, the topics needed to be studied and grouped into similar recurrent themes. This was done by independent researchers in order to reduce any potential bias when grouping themes. All topics were analysed by at least two researchers who had to agree that the topics should be grouped together. This reduced the list down to 38 major research themes. In our second survey we asked people to rank these 38 themes in order of importance. In the second survey, 460 people ranked the topics and identified which topics were the greatest priority.

**Research Priorities for Ireland**

The top RMD research priorities identified were:

- how to prevent RMDs from getting worse,
- how to diagnosis RMDs early and what impact an earlier diagnosis would have,
- how to improve pain management.

Other top priorities include the development of new treatments, improved understanding of RMDs in order to treat them better, and the role of genetics in RMDs. The interaction of RMDs with diet, exercise and mental health were also among the top priorities for RMD research.

**Why this is important**

It is hard to know what the community of people living with and supporting people with RMDs in Ireland truly need, and how to best meet those needs, if we do not ask. The participatory nature of the research priority setting allowed us to better understand the needs of the community as a whole. Focusing on what the most pressing issues for the Irish community can help us to better focus energy and resources towards research for the largest impact on people’s lives. Having a focal point can help us make real breakthroughs in our understanding of these complex issues.
